# Developing a Diagnostic Model to Predict the Risk of Asthma Based on Ten Macrophage-Related Gene Signatures

**DOI:** 10.1155/2022/3439010

**Published:** 2022-11-23

**Authors:** Xiaoshun Ai, Hong Shen, Yangyanqiu Wang, Jing Zhuang, Yani Zhou, Furong Niu, Qing Zhou

**Affiliations:** ^1^Huzhou First Hospital, Zhebei Mingzhou Hospital, No. 225, Gongyuan Road, Wuxing District, Huzhou Zhejiang Province, China 313000; ^2^School of Medicine, Huzhou University, No. 759 Erhuan East Road, Huzhou, Zhejiang Province, China 313000; ^3^Huzhou Hospital of Zhejiang University, Affiliated Central Hospital Huzhou University, No.1558, Sanhuan North Road, Wuxing District, Huzhou, Zhejiang Province, China 313000; ^4^Huzhou Central Hospital, Affiliated Central Hospital Huzhou University, Key Laboratory of Multiomics Research and Clinical Transformation of Digestive Cancer of Huzhou, No. 1558, Sanhuan North Road, Wuxing District, Huzhou Zhejiang Province, China 313000

## Abstract

**Objective:**

Asthma (AS) is a chronic inflammatory disease of the airway, and macrophages contribute to AS remodeling. Our study aims at screening macrophage-related gene signatures to build a risk prediction model and explore its predictive abilities in AS diagnosis.

**Methods:**

Three microarray datasets were downloaded from the GEO database. The Limma package was used to screen differentially expressed genes (DEGs) between AS and controls. The ssGSEA algorithm was used to determine immune cell proportions. The Pearson correlation coefficient was computed to select the macrophage-related DEGs. The LASSO and RFE algorithms were implemented to filter the macrophage-related DEG signatures to establish a risk prediction model. Receiver operating characteristic (ROC) curves were used to assess the diagnostic ability of the prediction model. Finally, the qPCR was used to detect the expression of selected differential genes in sputum from healthy people and asthmatic patients.

**Results:**

We obtained 1,189 DEGs between AS and controls from the combined datasets. By evaluating immune cell proportions, macrophages showed a significant difference between the two groups, and 439 DEGs were found to be associated with macrophages. These genes were mainly enriched in the gene ontology-biological process of immune and inflammatory responses, as well as in the KEGG pathways of cytokine-cytokine receptor interaction and biosynthesis of antibiotics. Finally, 10 macrophage-related DEG signatures *(EARS2*, *ATP2A2*, *COLGALT1*, *GART*, *WNT5A*, *AK5*, *ZBTB16*, *CCL17*, *ADORA3*, and *CXCR4*) were screened as an optimized gene set to predict AS diagnosis, and they showed diagnostic abilities with AUCs of 0.968 and 0.875 in ROC curves of combined and validation datasets, respectively. The mRNA expressions of *EARS2*, *ATP2A2*, *COLGALT1*, and *GART* in the control group were higher than in AS group, while the expressions of *WNT5A*, *AK5*, *ZBTB16*, *CCL17*, *ADORA3*, and *CXCR4* in the control group were lower than that in the AS group.

**Conclusion:**

We proposed a diagnostic model based on 10 macrophage-related genes to predict AS risk.\.

## 1. Introduction

Asthma (AS) is a respiratory disease that clinically manifests as airway hyperresponsiveness, inflammation, and mucus secretion [1]. As a heterogeneous clinical syndrome, AS affects more than 300 million people worldwide, with an annually increasing prevalence rate [2]. Environmental factors, comorbidities, genomic factors, and other social determinants are considered to play a synergistic role in the etiology of asthma [3]. Studies on the population genetics of AS have shown that genetic factors contribute to AS pathogenesis, with estimates of heritability ranging from 35 to 95% [4]. Therefore, it is necessary to study the candidate genes related to AS and AS phenotypes as well as their internal molecular mechanisms. Airway and alveolar epithelial cells are the first line of defense of the lung's immune system, protecting against invading pathogens and environmental pollutants [5]. AS is characterized by airway inflammation, and immune cell infiltration and goblet cell proliferation are often observed in the airways of AS patients [6]. The relationship between inflammatory response, immune activation, and AS exacerbation is well established; thus, studies on biomarkers associated with immune response in AS may help to improve clinical outcomes of AS by reducing early inflammation.

Macrophages contribute the largest proportion of leukocytes (accounting for approximately 70% of immune cells) found in alveoli, distal airspaces, and conducting airways [7, 8]. Macrophages are variously involved in AS inflammation, including altering the production of anti-inflammatory cytokines or chemokines and inducing inflammasomes to regulate cellular processes [9]. Although macrophages are abundant in lung tissues, their contribution to AS pathology comes more from functional changes. Studies have reported that macrophage function depends on the polarization state of Th1 and Th2 [10]. M1 macrophages are induced by IFN-*γ* and lipopolysaccharide and function in driving inflammation in response to intracellular pathogens, whereas M2 macrophages are involved in anti-inflammation induced by IL-4 and IL-13 [11]. These findings support the role of macrophages as disease modifiers, biomarkers, and therapeutic targets in AS. However, studies are still needed to explore how cellular signaling and gene signature expression influence the functional response of macrophages.

Therefore, the purpose of this study was to screen differentially expressed genes (DEGs) related to macrophages in AS through samples sourced from a public database. Then, macrophage-related DEG signatures were filtered as an optimized gene set to build a risk prediction model. Finally, the expression of candidate genes and the prediction model's efficiency in AS diagnosis and recognition were validated. The analytical flowchart of this study is summarized in Supplemental Figure 1. This study proposed novel biomarkers associated with macrophages in AS and provides new insights into AS therapeutic strategies by highlighting the potential function of macrophages.

## 2. Methods

### 2.1. Data Acquisition

The expression data were obtained from three microarray datasets in the Gene Expression Omnibus (GEO) (https://www.ncbi.nlm.nih.gov/) [12], including GSE137268, GSE148004, and GSE112260. In detail, 54 AS and 15 normal samples detected by the Illumina humanRef-8 v2.0 expression beadchip were collected from GSE63067; 9 AS and 9 normal samples detected by the Agilent-026652 Whole Human Genome Microarray 4 × 44 K v2 were obtained from GSE148004 [13], and 4 AS samples and 4 healthy controls were obtained from GSE112260 [14] based on the Affymetrix Human Gene 2.1 ST Array. Among them, GSE137268 and GSE148004 were utilized for analysis, while GSE112260 was used for validation.

### 2.2. Screening for DEGs of AS

Principal component analysis (PCA) was performed to remove the batch effect of samples in GSE137268 and GSE148004 using the R3.6.1 sva package version 3.38.0 [15] (http://www.bioconductor.org/packages/release/bioc/html/sva.html). After gathering the combined expression profile data, the R3.6.1 Limma package version 3.34.7 [16] (https://bioconductor.org/packages/release/bioc/html/limma.html) was used to screen the DEGs of AS with a standard of fold discovery rate (FDR) < 0.05, and |log2fold change (FC)| > 0.263.

### 2.3. Screening of Macrophage-Related DEGs

To evaluate immune cell infiltration in the combined samples, immunologic signature gene sets were downloaded from the Gene Set Enrichment Analysis website (GSEA, http://software.broadinstitute.org/gsea/index.jsp). After this, single-sample gene set enrichment analysis (ssGSEA) [17] was implemented using the gene set variation analysis (GSVA) package version 1.36.3 [18] (http://www.bioconductor.org/packages/release/bioc/html/GSVA.html) in R3.6.1, to compare the differences in the proportion of individual immune cells between AS and normal samples.

The correlation between the DEGs of AS and the proportion of macrophages was assessed using the R3.6.1 cor function (http://77.66.12.57/R-help/cor.test.html). By calculating the Pearson correlation coefficient (PCC), DEGs with *P* < 0.05 were determined to be significantly associated with macrophages.

The R package clusterProfiler (http://bioconductor.org/packages/release/bioc/html/clusterProfiler.html) was used for analysis of gene ontology biological process (GO-BP) function and Kyoto Encyclopedia of Genes and Genomes (KEGG) pathway enrichment of macrophage-related DEGs. The *P* adjust less than 0.05 and the count value was greater than 1 were considered as the threshold screening criterion.

### 2.4. Construction of Protein-Protein Interaction (PPI) Network

STRING version 11.0 [19] (http://string-db.org/) was used to establish the interactions of the coding proteins of macrophage-related DEGs with a combined score threshold of 0.6. A PPI network was constructed and visualized using Cytoscape version 3.6.1 [20] (http://www.cytoscape.org/). KEGG pathway enrichment analysis of hub genes in the network was then performed using the R package clusterProfiler.

### 2.5. Screening and Verification of Macrophage-Related Gene Signatures

Two different algorithms were used to screen the DEG signatures from hub genes in the PPI network. Specifically, the lars package version 1.2 [21] (https://cran.r-project.org/web/packages/lars/index.html) was used for least absolute shrinkage and selection operator (LASSO) regression analysis on hub genes, and the caret package version 6.0-76 [22] (https://cran.r-project.org/web/packages/caret) was used for recursive feature elimination (RFE) to select candidate genes. The intersection DEGs were then determined to be macrophage-related DEG signatures.

The expression data of macrophage-related DEG signatures were extracted from the GSE112260 dataset and compared between AS and the controls. The support vector machine (SVM) approach [23] was utilized to construct a disease diagnostic classifier (Core: Sigmoid Kernel; Cross: 100-fold cross-validation) using R3.6.1 e1071 version 1.6-8 (https://cran.r-project.org/web/packages/e1071). The sensitivity and specificity of the receiver operating characteristic (ROC) curve calculated using R 3.6.1 pROC version 1.12.1 [24] (https://cran.r-project.org/web/packages/pROC/index.html) were used to evaluate the performance of the diagnostic model in the combined dataset and validation dataset.

The patients with AS admitted to the Huzhou Traditional Chinese Medicine Hospital and healthy volunteers from the physical examination center in Huzhou Traditional Chinese Medicine Hospital were recruited in the study as subjects. The Ethics Committee of Huzhou Traditional Chinese Medicine Hospital approved this study, and all subjects provided informed consent (N0.2021-030-A) for approval. These are the inclusion criteria: subjects were diagnosed with AS according to National Asthma Education and Prevention Program Coordinating Committee Expert Panel Working Group (NAEPP). The exclusion criteria are as follows: subjects with other respiratory diseases, such as allergic rhinitis, endotracheal disease, bronchial lung cancer, etc. Patients with other malignant tumors or serious cardiopulmonary diseases. The patients had taken bronchodilators, glucocorticoids, and other asthma medications a week earlier. Sputum from asthmatic patients was obtained as follows. After inhalation of hypertonic saline atomization for 15 min, the lungs were tapped, and sputum was extracted and collected in a sterile environment. The clinical characteristics of the patients are shown in Supplemental Table 1.

### 2.6. qPCR Analysis

TRIzol (Invitrogen; Thermo Fisher Scientic, Inc) was added to the cells after washing with PBS. RNA was extracted according to a previously described method. The quality and concentration of RNA were measured by an Infinite M100 PRO (Tecan Group Ltd., China). cDNA was obtained using RRO47A (TAKARA BIO INC, Japan) according to the protocols. Subsequently, real-time qPCR (Funglyn Biotech, Inc, Ontario, Canada) was performed at 50°C for 3 min, followed by 95°C for 3 min, 95°C for 10 s, and 60°C for 30 s; this was repeated for 40 cycles. Information on primers is shown in Supplemental Table 2. The expression levels of miRNAs and genes were measured using the 2^−△△Ct^ method.

## 3. Results

### 3.1. Screening of DEGs between AS Samples and Healthy Controls

**Figure 1:**
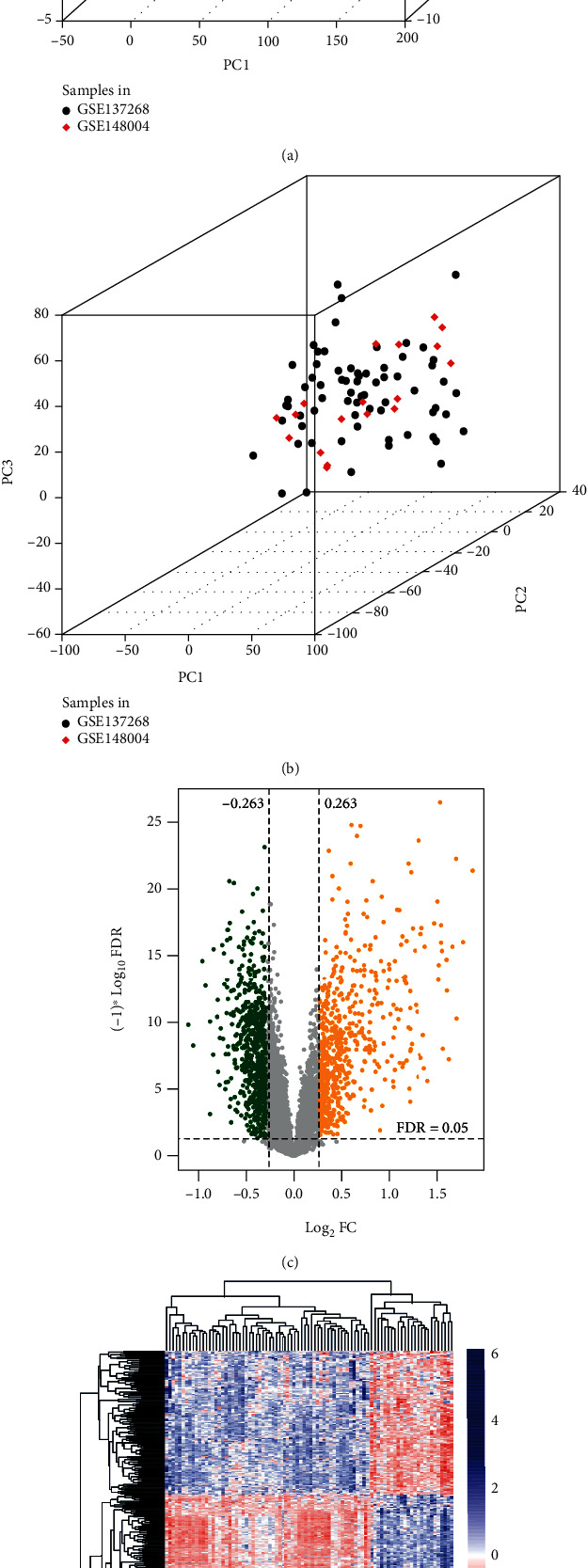
Screening of DEGs between AS and normal samples. (a, b) PCA diagrams before and after removing the batch effect on samples from the two datasets. Black and red dots represent samples in GSE137268 and GSE148004, respectively. (c) The volcano plot showed 1,189 DEGs between AS and the control groups screened from combined samples with the FDR < 0.05 and |log2FC| > 0.263 criteria. The *x*-axis indicates the value of log_2_FC, while the *y*-axis indicates the FDR value. The green and orange dots represent downregulated and upregulated DEGs, respectively, with statistical significance. (d) Heatmap based on the expression levels of DEGs.

We combined GSE137268 and GSE148004 into one dataset and removed the batch effect using the SVA algorithm. The expression levels before and after removal of the batch effect are shown in Supplemental Figure 2. A PCA of the combined samples was further performed (Figures 1(a) and 1(b)), and the results indicated that the combined samples from the two different detection platforms were indistinguishable after batch effect removal and could be applied for further analysis. The Limma package was then used to screen DEGs between AS samples and healthy controls from the combined dataset, and we obtained 1,189 DEGs in total with the corresponding thresholds, as shown in Figure 1(c). The heatmap (Figure 1(d)) showed that DEG expression was significantly different between the AS and control groups, indicating that the screened DEGs had expression features in each group.

### 3.2. Selection of Macrophage-Related DEGs and Functional Enrichment Analysis

**Figure 2:**
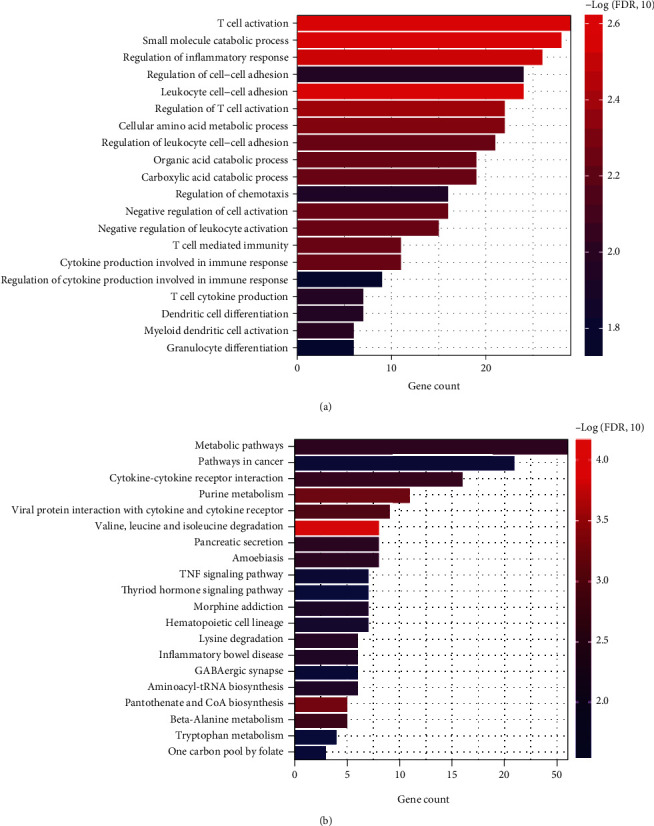
Functional analysis and pathway enrichment of macrophage-related DEGs. (a, b) Enriched GO-BP and KEGG pathways of macrophage-related DEGs with statistical significance. The *x*-axis indicates the gene count; the *y*-axis indicates the terms of biological processes or pathways, and the colors indicate the -log *P* value.

Based on the expression profiling of the combined samples, we obtained the proportions of 28 types of immune cells using the ssGSEA algorithm. By comparing the differences in the proportion of immune cells between AS samples and healthy controls, we found that seven immune cell types were significantly different between the two groups, including macrophages, with a *P* value of 0.011 (Supplemental Figure 3 and Supplemental Table 3). By calculating the PCC between DEGs and cell proportion of macrophages in samples using the cor function in R3.6.1, 439 macrophage-related DEGs were screened at a *P* value < 0.05. We then carried out function and pathway enrichment analyses on macrophage-related DEGs using the R package clusterProfiler and filtered 20 biological processes (Figure 2(a)), including regulation of inflammatory response as well as 20 KEGG pathways (Figure 2(b)) including Metabolic pathways, pathways in cancer and cytokine-cytokine receptor interaction, etc., with statistical significance.

### 3.3. Establishment and Analysis of PPI Network

**Figure 3:**
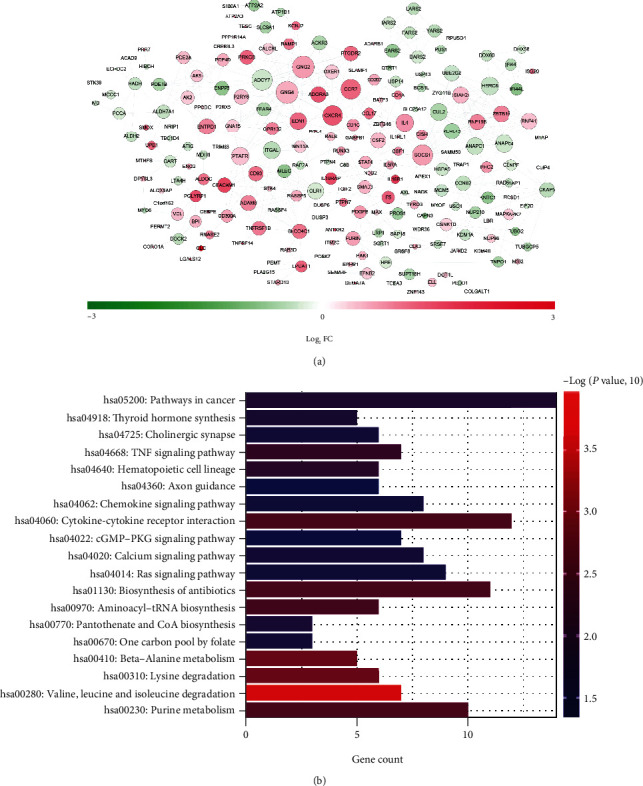
PPI network construction and pathway enrichment of genes in the network. (a) Interactions between the coding proteins of the macrophage-related DEGs. The changes from green to red of nodes indicate the downregulation-upregulation of macrophage-related DEGs, and the sizes of nodes indicate the degree of connection in the network. (b) KEGG pathway enrichment of genes in the PPI network. The *x*-axis indicates the gene count; the *y*-axis indicates the terms of KEGG pathways, and the colors indicate the -log *P* value.

The STRING database was applied to establish the interactions between the coding proteins of macrophage-related DEGs, and a total of 515 pairs of interactions were obtained with combined scores over 0.6. A PPI network comprising 220 gene nodes was constructed, as shown in Figure 3(a). We also analyzed the topology properties of nodes in the network and listed the detailed information of nodes with degrees > 10 in Supplemental Table 4. Furthermore, pathway enrichment was performed based on 220 macrophage-related DEGs in the PPI network (Figure 3(b)); nineteen KEGG pathways were emphasized, including cancer, cytokine-cytokine receptor interaction, biosynthesis of antibiotics, and purine metabolism pathways, among others.

### 3.4. Screening of Macrophage-Related DEG Signatures to Build a Risk Prediction Model

**Figure 4:**
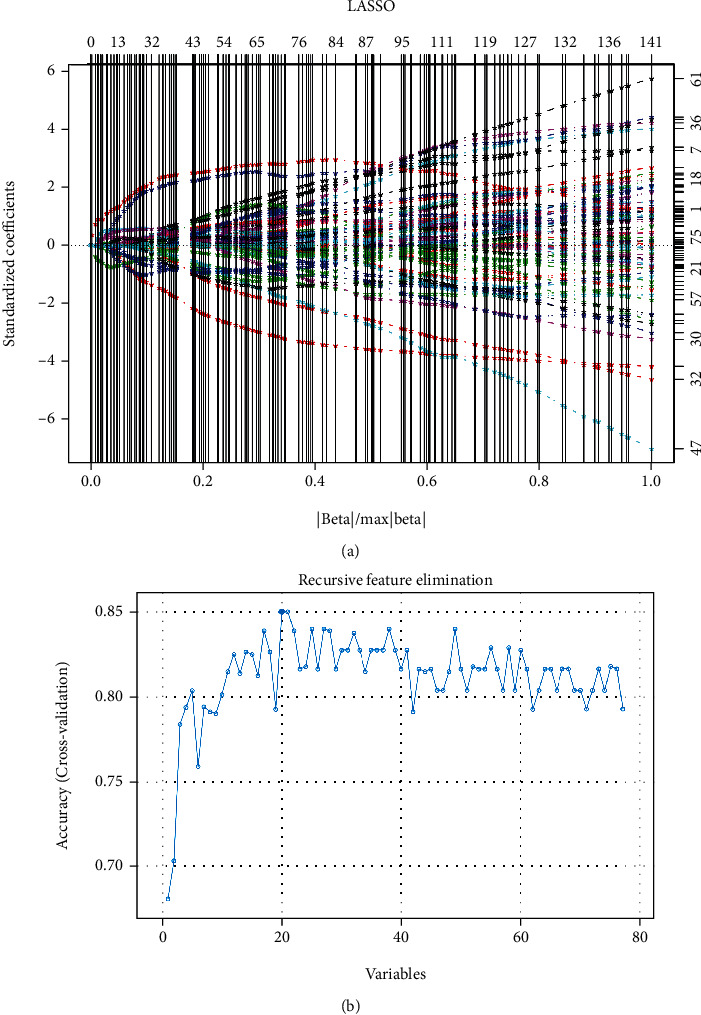
(a, b) Parameter diagrams of LASSO and RFE algorithms. (a) LASSO regression analysis provided variables with nonzero coefficients under different parameters. Below the *x*-axis are the values of different parameters in the combined dataset, and above the *x*-axis are the numbers of variables with the corresponding parameter. The left *y*-axis represents variable coefficients under different parameters, and the right *y*-axis represents the number of nonzero coefficient variables with the corresponding parameter. (b) The RFE algorithm provided a combination of variables with the highest accuracy of the prediction model. The *x*-axis indicates the number of variables, and the *y*-axis indicates the accuracy of the prediction model.

A total of 77 macrophage-related DEGs from the PPI network were found to be enriched in the KEGG pathways, and LASSO and RFE were implemented to further screen out DEG signatures to establish a diagnostic model with more accurate predictions. The parameter diagrams of LASSO and RFE are shown in Figure 4. Through LASSO regression analysis, 14 DEGs were found to have significant predictive advantages. Moreover, the RFE algorithm provided a combination of 20 DEGs as a stable prediction feature. Considering the intersection of the LASSO regression analysis and RFE algorithm, 10 macrophage-related DEG signatures (*EARS2*, *ATP2A2*, *COLGALT1*, *GART*, *WNT5A*, *AK5*, *ZBTB16*, *CCL17*, *ADORA3*, and *CXCR4*) were finally selected as an optimized gene set to predict AS diagnosis. Within the optimized gene set, *CXCR4*, *ZBTB16*, and *ADORA3* had relatively higher degrees of connection in the PPI network.

### 3.5. Validation of Gene Expression and Prediction Model Efficacy

**Figure 5:**
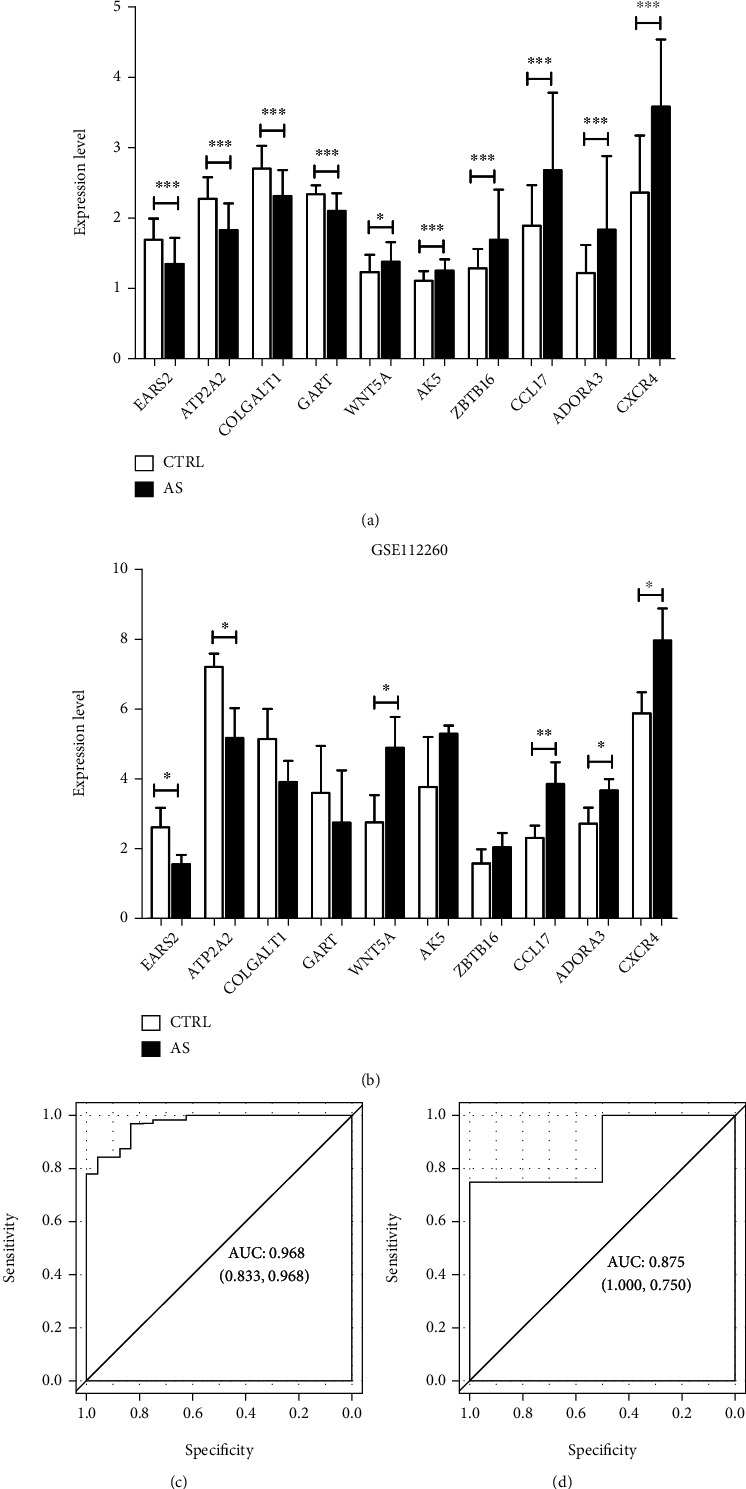
Expression validation of macrophage-related DEG signatures and performance validation of the diagnostic model. (a, b) Expression differences of 10 macrophage-related DEG signatures between the AS and control groups in the combined dataset and GSE112260. The *x*-axis indicates the 10 DEG signatures, and the *y*-axis indicates the expression level. ^∗^*P* < 0.05, 0.005 <  ^∗∗^*P* < 0.05, ^∗∗∗^*P* < 0.005. (c, d) ROC curves showing the abilities of diagnostic models in the combined dataset and GSE112260 with AUCs of 0.968 and 0.875, respectively. The *x*-axis indicates the value of specificity, and the *y*-axis indicates the value of sensitivity.

The expression data of the 10 macrophage-related DEG signatures were extracted and compared between AS samples and healthy controls in both the combined and GSE112260 datasets. As shown in Figure 5(a), the expression of all 10 DEG signatures was significantly different between the two groups in the combined dataset. Meanwhile, in the GSE112260 dataset (Figure 5(b)), the expression trends of the 10 macrophage-related DEGs were in accordance with those in the combined dataset, and the expression differences of *EARS2*, *ATP2A2*, *WNT5A*, *CCL17*, *ADORA3*, and *CXCR4* were significant between the AS and control groups (*P* < 0.05).

Based on the 10 macrophage-related DEG signatures, we constructed a diagnostic model using the combined dataset and validated model performance using the GSE112260 dataset. ROC curves showed excellent predictive abilities of diagnostic models with an area under the curve (AUC) of 0.968 and 0.875 in the combined and GSE112260 datasets, respectively.

### 3.6. Verification of 10 Differential Genes by qPCR in Clinical Samples

**Figure 6:**
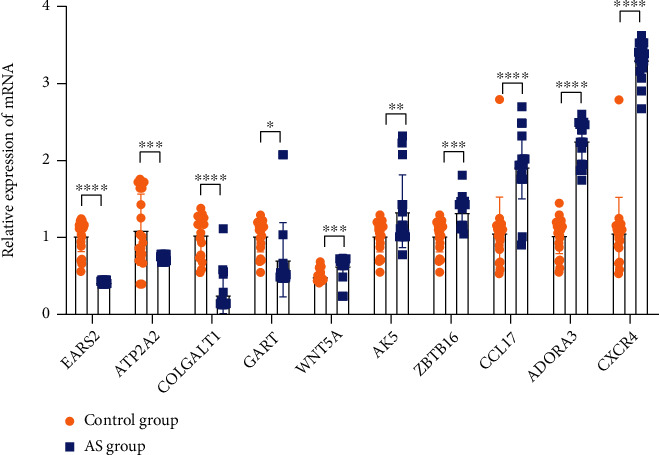
Verification of 10 differential genes by qPCR in clinical samples. The 10 macrophage-related DEG signatures (*EARS2, ATP2A2, COLGALT1, GART, WNT5A, AK5, ZBTB16, CCL17, ADORA3,* and *CXCR4*) were verified by qPCR.. ^∗^*P* < 0.05, 0.005 <  ^∗∗^*P* < 0.05, ^∗∗∗^*P* < 0.005, ^∗∗∗∗^*P* < 0.001.

The 10 macrophage-related DEG signatures (*EARS2*, *ATP2A2*, *COLGALT1*, *GART*, *WNT5A*, *AK5*, *ZBTB16*, *CCL17*, *ADORA3*, and *CXCR4*) were verified by qPCR. The results showed that the mRNA expressions of *EARS2*, *ATP2A2, COLGALT1*, and *GART* in the control group (healthy volunteers) were higher than in AS group, while the expressions of *WNT5A*, *AK5*, *ZBTB16*, *CCL17*, *ADORA3*, and *CXCR4* in control group were lower than that in the AS group (Figure 6).

## 4. Discussion

AS is a multifaceted disease that affects all age groups, and genetic factors play an important role in the risk of developing AS. Although numerous genes, including *ORMDL3* and *GSDB* at locus 17q21 have been shown to contribute to the genetic etiology of AS [25, 26], there is a lack of strategies for integrating risk factors with multiple datasets to establish relationships among genetics, immunity, and AS. In this study, we first screened 1,189 DEGs from 63 AS and 24 control samples from the combined dataset. Macrophages were found to be significantly different between the two groups, and 439 macrophage-related DEGs were further screened after establishing the relationship between DEGs and the abundance of macrophage immune infiltration. These macrophage-related DEGs were mainly enriched in the GO-BP of immune and inflammatory responses as well as KEGG pathways of cytokine-cytokine receptor interaction and biosynthesis of antibiotics. By applying the LASSO and RFE algorithms, we finally screened 10 macrophage-related DEG signatures to establish a risk prediction model, which was shown to have excellent AS diagnostic abilities with AUCs of 0.968 and 0.875 in the ROC curves of the combined and GSE112260 datasets, respectively. Our findings propose significant biomarkers for AS diagnosis driven by macrophages.

AS is known to be associated with immune system activation, and both innate and adaptive immunity play roles in the immune mechanism of AS [27]. In this study, we found seven types of immune cells (including macrophages, natural killer cells, and immature dendritic cells, among others) that were significantly different between AS samples and healthy controls using a ssGSEA algorithm. Studies have shown that polarized M1/M2 macrophages function as antigen-presenting cells that may effectively activate Th1, Th2, Th17, or Treg cells in AS [28]. Moreover, a related study pointed out that the role of natural killer cells in the regression of peribronchial cell infiltration in AS may be to inhibit antigen-specific Th17 and Th1 immunity [29]. Additionally, dendritic cells are the most potent antigen-presenting cells in the immune system and play a central role in the allergen-driven Th2 immune response in AS [30]. This may explain the possible regulatory mechanism of macrophages with the combined action of natural killer cells and dendritic cells in activating Th1, Th2, and Th17 immune responses in AS attacks.

By evaluating the relationship between DEGs in AS and the proportion of macrophages, we obtained 439 macrophage-related DEGs that were mainly enriched in the biological processes of immune and inflammatory responses. Saradna et al. concluded that the regulation process of macrophages contained intricate interactions among various cytokines, chemokines, transcription factors, and immunomodulatory cells [11]; our results provide potential targets for such a complex interplay in macrophages and related inflammatory responses in AS. These macrophage-related DEGs were also enriched in the KEGG pathways of cytokine-cytokine receptor interactions. Zhao et al. found that the genes expressed in macrophages under hypoxic conditions were enriched in the cytokine-cytokine receptor interaction pathway [31]. Moreover, upregulated DEGs in acute respiratory distress syndrome were also determined to be associated with cytokine-cytokine receptor interactions [32]. Combined with the above findings, we hypothesized that dyspnea and hypoxia caused by asthma attacks might induce differential expression of these macrophage-related genes, which are closely related to the inflammatory and immune responses and cytokine-cytokine receptor interaction.

In the present study, LASSO and RFE algorithms were applied to establish a diagnostic model with more accurate predictions. LASSO analysis is a high-dimensional predictive regression method that integrates multiple biomarkers into a single model to enhance their predictive value [33]. In their prediction of miRNA-mRNA relationships in prostate cancer, Lu et al. identified LASSO as an informative tool in constructing diagnostic models with considerable advantages in sensitivity and specificity [34]. Kim SM and Kim Y also proposed that the prediction performance of LASSO regression for disease diagnosis was higher than that of stepwise logistic regression [35]. Additionally, SVM-RFE has been identified as an effective feature selection algorithm for feature screening in complex high-dimensional biological data and has been widely used in disease research and drug development [36]. Furthermore, Sanz et al. stated that in biomedical data analysis, RFE could accurately select variables and assess the direction and strength of associations [37]. The high specificity and sensitivity of the ROC curves in this study indicated that LASSO and RFE algorithms are superior in screening gene signatures to predict AS risk.

By taking the intersection of LASSO and RFE, we finally obtained 10 macrophage-related DEG signatures to predict AS risk and identify disease diagnostic performance. Among these candidate genes, the expression of *EARS2*, *ATP2A2*, *WNT5A*, *CCL17*, *ADORA3*, and *CXCR4* was further validated in both the combined and GSE112260 datasets. During the construction of a mouse model with conditional loss of function, Li et al. demonstrated that differentiation and migration of myofibroblasts were the main effects of *WNT5A* inactivation on alveolar formation [38]. Smooth muscle-derived *WNT5A* enhanced Th2 inflammation in AS, leading to increased airway wall inflammation and remodeling [39]. Moreover, Williams et al. reported that mice with human rhinovirus-induced AS could upregulate the expression of *CCL17* by activating lung STAT6 [40]. However, Yuan et al. found that the deficiency of integrin *β*4 was involved in the Th2 response in allergic AS by downregulating the *CCL17* pathway in airway epithelial cells [41]. The key roles of these feature genes in AS have been elucidated, but the molecular mechanism by which they mediate AS inflammation through macrophages is still unclear.

In this study, we screened gene signatures associated with macrophages and investigated their role in the diagnosis of AS. It is not only important for the diagnosis of asthma but also necessary to study the mechanism of asthma. It is important to further study the expression of these genes signatures in peripheral blood samples and tissue samples.

Although we found several interesting macrophage-related gene signatures and explored their roles in AS diagnosis, the inability to define the relationship between candidate genes and the severity and clinical AS phenotype caused by the lack of clinical information on samples was one of the limitations of the present study. In addition, the lack of multicenter large sample verification is also the deficiency of this study. In future studies, more solid tumor samples should be collected to verify the differences in the expression of these candidate genes and further explore their regulatory mechanisms in the inflammatory response and immune activation of AS.

## 5. Conclusion

Here, we obtained 439 DEGs in AS associated with macrophages, which also significantly differed in cell proportion between AS and the controls. LASSO and RFE algorithms that can effectively identify and screen disease characteristic variables were then employed, and 10 macrophage-related DEG signatures were ultimately screened to establish a risk prediction model of AS by considering the intersection of relevant results. This prediction model showed excellent AS diagnostic abilities, with high sensitivity and specificity. Our findings may help to better understand the mechanisms of macrophage-mediated regulation in the pathogenesis of AS and provide potential diagnostic biomarkers for patients with AS.

## Data Availability

The datasets generated during the current study are not publicly available but obtained from corresponding authors on reasonable request.

## Abbreviations

AS: Asthma
DEGs: Differentially expressed genes
GEO: Gene expression omnibus
PCA: Principal component analysis
FDR: Fold discovery rate
FC: Fold change
GSEA: Gene set enrichment analysis
ssGSEA: Single sample gene set enrichment analysis
GSVA: Gene set variation analysis
PCC: Pearson correlation coefficient
GO-BP: Gene ontology-biology process
KEGG: Kyoto encyclopedia of genes and genomes
PPI: Protein-protein interaction
LASSO: Least absolute shrinkage and selection operator
RFE: Recursive feature elimination
SVM: Support vector machine
ROC: Receiver operating characteristic
AUC: Area under curve.

## Additional Points

*Highlights.* (1) We found 439 macrophage-related DEGs involved in the inflammation response of AS. (2) Macrophages showed significant differences in cell proportion between AS and controls. (3) LASSO and RFE were used to select gene signatures for building a risk prediction model. (4) Ten macrophage-related DEG signatures showed excellent AS diagnostic abilities. (5) The mRNA expressions of *EARS2*, *ATP2A2*, *COLGALT1*, and *GART* in the control group were higher than AS group, while the expressions of *WNT5A, AK5, ZBTB16*, *CCL17*, *ADORA3*, and *CXCR4* in the control group were lower than that in the AS group.

## Supplementary Materials

Supplementary 1 Supplemental Figure 1: the study flowchart.

Supplementary 2 Supplemental Figure 2: expression levels of samples (a) before and (b) after SVA batch effects.

Supplementary 3 Supplemental Figure 3: immune cells with significant differences in cell proportion between AS samples and controls. The *x*-axis indicates different kinds of immune cells with statistical significance, and the *y*-axis indicates the value of the cell fraction.

Supplementary 4 Supplemental Table 1: basic information of CRC samples.

Supplementary 5 Supplemental Table 2: primers of genes used for qPCR.

Supplementary 6 Supplemental Table 3: differences in proportion of twenty-eight types of immune cells between AS and controls.

Supplementary 7 Supplemental Table 4: nodes with degrees of connection over ten in the PPI network.
