# Supplementary figures and images for: Developing a Diagnostic Model to Predict the Risk of Asthma Based on Ten Macrophage-Related Gene Signatures
Source: Biomed Res Int. 2022 Nov 23;2022:3439010. doi: 10.1155/2022/3439010 (PMC9713468; doi:10.1155/2022/3439010)

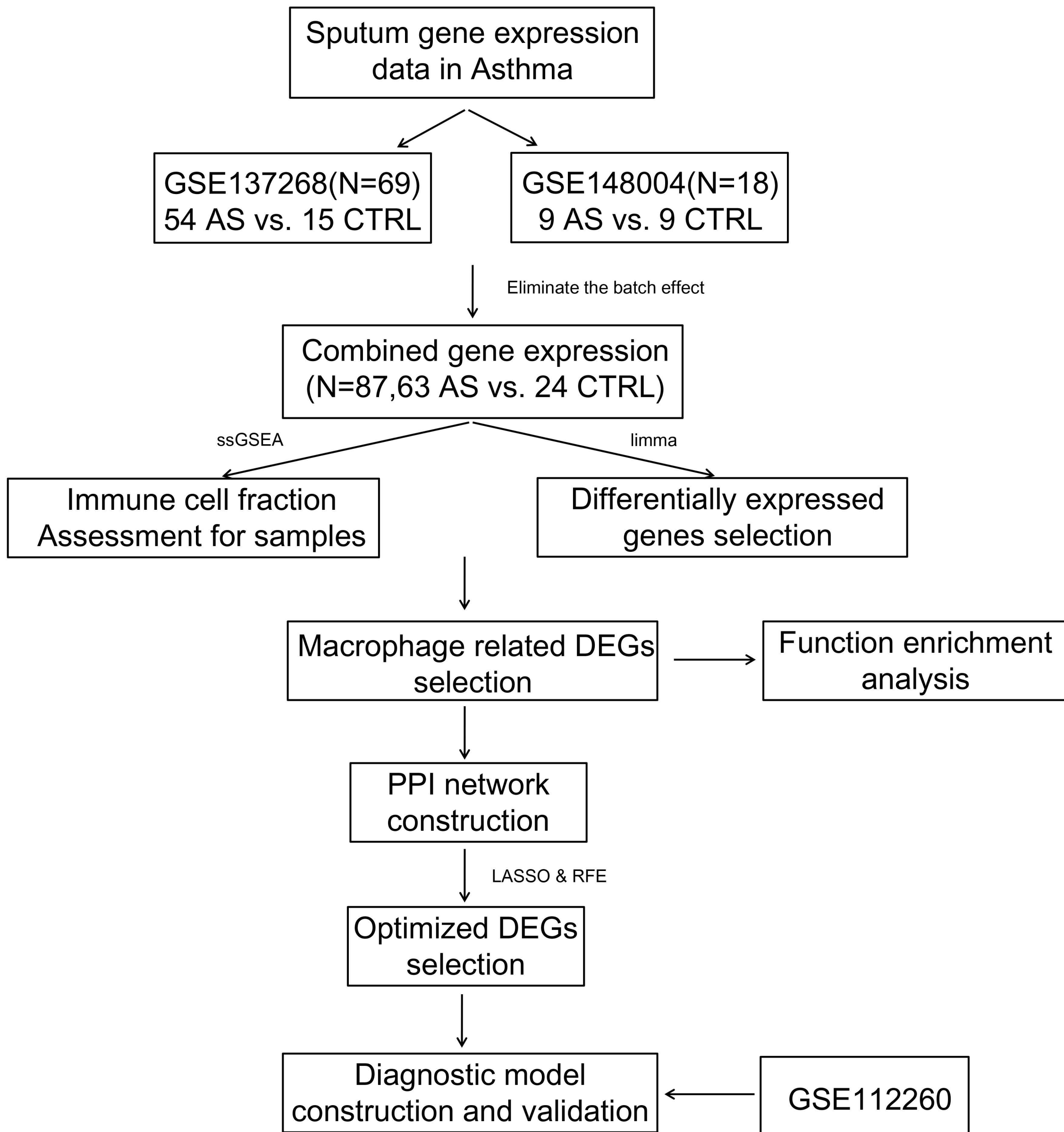

Supplement: Supplementary 1 — Supplemental Figure 1: the study flowchart. [file 3439010.f1.pdf]

**A****Before sva batch effect**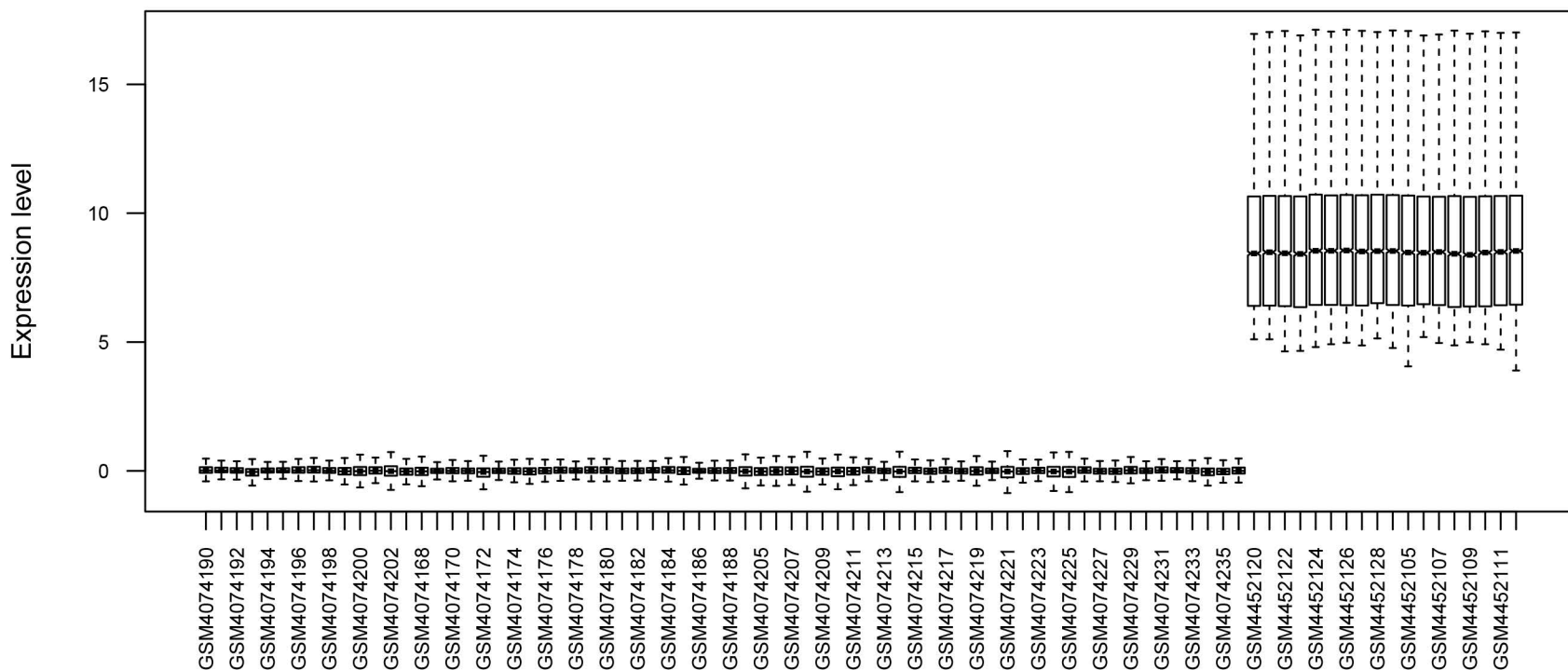**B****After sva batch effect**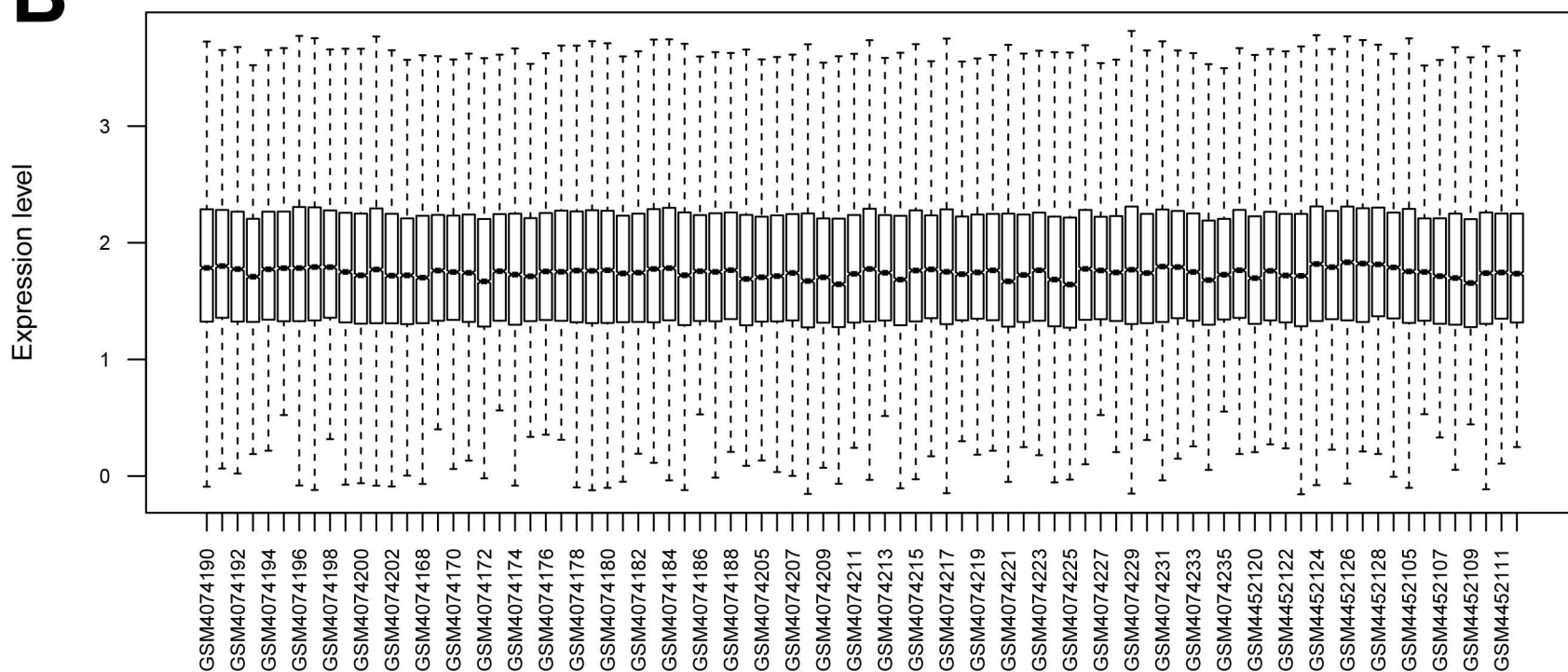

Supplement: Supplementary 2 — Supplemental Figure 2: expression levels of samples (a) before and (b) after SVA batch effects. [file 3439010.f2.pdf]

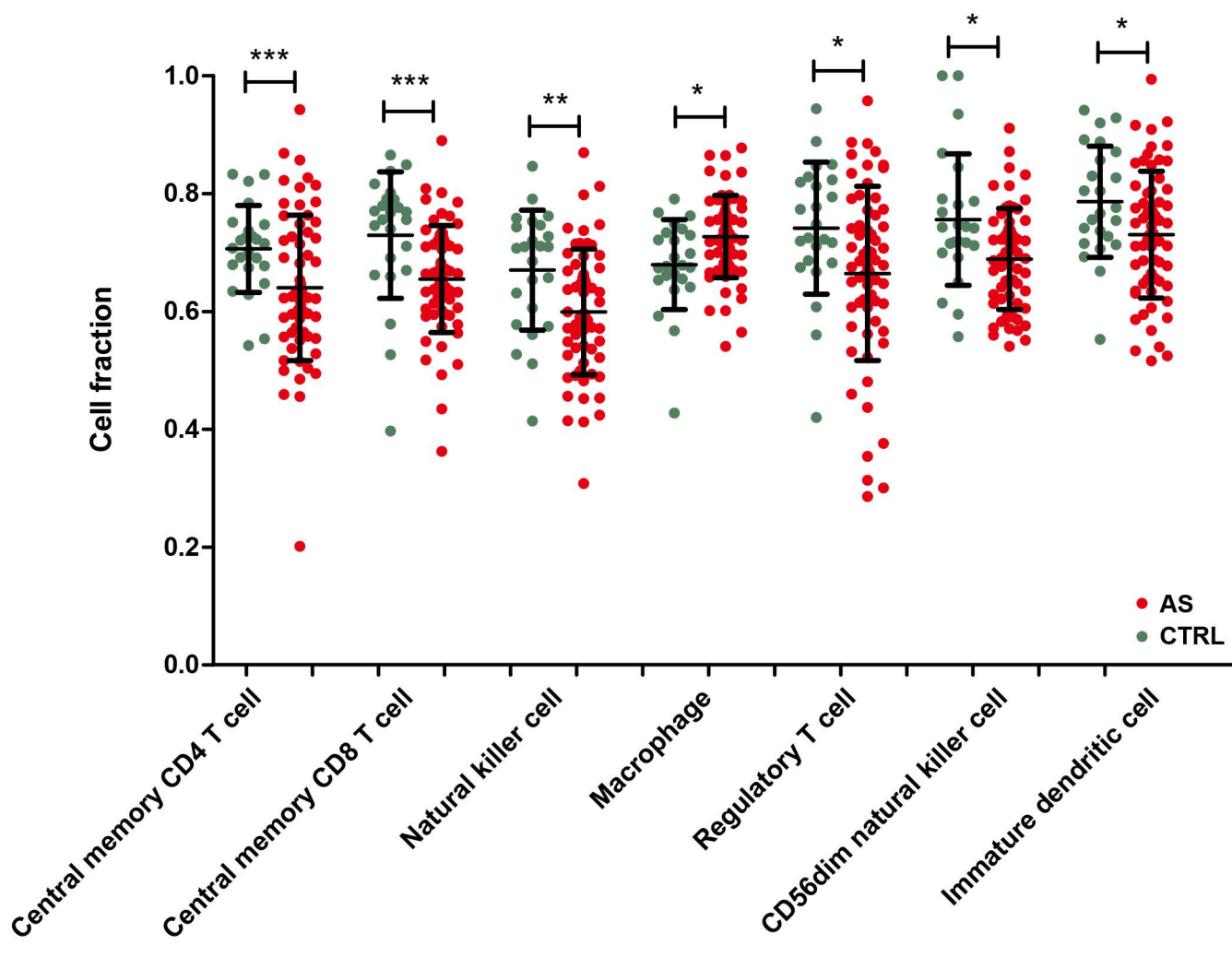

Supplement: Supplementary 3 — Supplemental Figure 3: immune cells with significant differences in cell proportion between AS samples and controls. The x-axis indicates different kinds of immune cells with statistical significance, and the y-axis indicates the value of the cell fraction. [file 3439010.f3.pdf]
