# Supplementary material for: Developing a Diagnostic Model to Predict the Risk of Asthma Based on Ten Macrophage-Related Gene Signatures
Source: Biomed Res Int. 2022 Nov 23;2022:3439010. doi: 10.1155/2022/3439010 (PMC9713468; doi:10.1155/2022/3439010)
Supplement: Supplementary 4 — Supplemental Table 1: basic information of CRC samples. [file 3439010.f4.docx]

**Supplemental Table 1 Basic information of samples**

|  | **Healthy volunteers** | **AS** | **P value** |
| --- | --- | --- | --- |
| Male, n | 8/20 | 6/20 | 0.507 |
| Age, years | 53.70±9.53 | 50.70±10.28 | 0.344 |
| Current smoking, n | 6/20 | 9/20 | 0.327 |
| Current drinking, n | 10/20 | 6/20 | 0.197 |
| Known diabetes, n | 4/20 | 5/20 | 0.705 |
| Known hypertension, n | 5/20 | 9/11 | 0.185 |
