# Supplementary material for: Developing a Diagnostic Model to Predict the Risk of Asthma Based on Ten Macrophage-Related Gene Signatures
Source: Biomed Res Int. 2022 Nov 23;2022:3439010. doi: 10.1155/2022/3439010 (PMC9713468; doi:10.1155/2022/3439010)
Supplement: Supplementary 5 — Supplemental Table 2: primers of genes used for qPCR. [file 3439010.f5.docx]

**Supplemental Table 2 Primers of genes used for qPCR**

|  | **Forward primer** | **Reverse primer** |
| --- | --- | --- |
| GAPDH | GGACTGACCTGCCGTCTAG | TAGCCCAGGATGCCCTTGAG |
| β-actin | CGTGACATTAAG GAGAAGCTG | CTAGAAGCATTTGCGGTGGAC |
| EARS2 | TCAAGTGAAGTGTGCTGTGC | AAACACTGGGGATGGGCTTG |
| ATP2A2 | CTCGGATCCAACACTACAGGTGTTGAATGG | CGGAATTCATGCGCAGTGATAAATTGAC |
| COLGALT1 | TGGCTACGGACCACAACATG | CCGCCTCAGGTTGATCATGA |
| GART | CCCUCAGGUUUCUAAUGAUT | AUCAUUAGAAACCUGAGGTT |
| WNT5A | CAACTGGCAGGACTTTCTCA | TTCTTTGATGCCTGTCTCG |
| AK5 | CGGGAATCTGACACAGGTGCT | GATCCGGACTACGATCGTTTTGT |
| ZBTB16 | GTCTCCATGGACTTCAGCAC | TACGTCTTCATCCCACTGTG |
| CCL17 | TCTGCCCGCTCAAACAGTTG | TCAAACAGTTGTGGTCGAAGGAGTCGG |
| ADORA3 | CCTGGGCATCACAATCCACT | ACCCTCTTGTATCTGACGGTA |
| CXCR4 | CCGTGGCAAACTGGTACTTT | GACGCCAACATAGACCACCT |
