# Supplementary material for: Developing a Diagnostic Model to Predict the Risk of Asthma Based on Ten Macrophage-Related Gene Signatures
Source: Biomed Res Int. 2022 Nov 23;2022:3439010. doi: 10.1155/2022/3439010 (PMC9713468; doi:10.1155/2022/3439010)
Supplement: Supplementary 6 — Supplemental Table 3: differences in proportion of twenty-eight types of immune cells between AS and controls. [file 3439010.f6.docx]

**Supplemental Table 3 Differences in proportion of twenty-eight types of immune cells between AS and controls**

| **Immune cells** | ***P*-value** |
| --- | --- |
| Central memory CD4 T cell | 0.003243966 |
| Central memory CD8 T cell | 0.004724623 |
| Natural killer cell | 0.006406344 |
| Macrophage | 0.011496729 |
| Regulatory T cell | 0.011628295 |
| CD56dim natural killer cell | 0.01209186 |
| Immature dendritic cell | 0.021927137 |
| Myeloid derived suppressor cell | 0.052926468 |
| Neutrophil | 0.058532052 |
| Effector memeory CD4 T cell | 0.088614339 |
| Type 17 T helper cell | 0.094973258 |
| Immature B cell | 0.105332358 |
| Gamma delta T cell | 0.123570864 |
| Mast cell | 0.154776068 |
| Natural killer T cell | 0.171657443 |
| Type 1 T helper cell | 0.221669837 |
| Activated CD8 T cell | 0.233180861 |
| T follicular helper cell | 0.23780204 |
| Eosinophil | 0.282423834 |
| Plasmacytoid dendritic cell | 0.33893601 |
| Activated dendritic cell | 0.402065855 |
| Activated B cell | 0.493047371 |
| CD56bright natural killer cell | 0.648101181 |
| Memory B cell | 0.708535729 |
| Monocyte | 0.731458055 |
| Effector memeory CD8 T cell | 0.755891002 |
| Type 2 T helper cell | 0.890413236 |
| Activated CD4 T cell | 0.976857793 |

Bold *P*<0.05 indicates statistical significance.
