# Supplementary material for: Developing a Diagnostic Model to Predict the Risk of Asthma Based on Ten Macrophage-Related Gene Signatures
Source: Biomed Res Int. 2022 Nov 23;2022:3439010. doi: 10.1155/2022/3439010 (PMC9713468; doi:10.1155/2022/3439010)
Supplement: Supplementary 7 — Supplemental Table 4: nodes with degrees of connection over ten in the PPI network. [file 3439010.f7.docx]

**Supplemental Table 4 Nodes with degrees of connection over ten in the PPI network**

| **Symbol** | **Average Shortest Path Length** | **Betweenness Centrality** | **Closeness Centrality** | **LogFC** | **Degree** |
| --- | --- | --- | --- | --- | --- |
| *GNG2* | 3.72272727 | 0.04743876 | 0.26862027 | 0.614986416 | 19 |
| *GNG4* | 3.72272727 | 0.04743876 | 0.26862027 | 0.495153458 | 19 |
| *SOCS1* | 3.74545455 | 0.19059545 | 0.26699029 | 0.601607758 | 17 |
| *ADCY7* | 3.76818182 | 0.1432983 | 0.26537998 | -0.302179956 | 17 |
| *CCR7* | 3.55 | 0.1406692 | 0.28169014 | 1.036865302 | 16 |
| *CXCR4* | 3.62272727 | 0.18133521 | 0.27603513 | 1.195617008 | 16 |
| *HERC6* | 4.40909091 | 0.04462433 | 0.22680412 | -0.349502388 | 13 |
| *ANAPC4* | 4.24545455 | 0.05479496 | 0.23554604 | -0.305391862 | 13 |
| *PTAFR* | 4.13636364 | 0.04613461 | 0.24175824 | 0.351371482 | 13 |
| *IL4* | 3.79090909 | 0.08352055 | 0.26378897 | 0.696198098 | 13 |
| *ANAPC1* | 4.27727273 | 0.03115412 | 0.23379384 | -0.341756414 | 12 |
| *ITGAL* | 3.75454545 | 0.13093529 | 0.26634383 | -0.4183165 | 12 |
| *EDN1* | 3.72272727 | 0.0776658 | 0.26862027 | 1.00629191 | 12 |
| *CUL2* | 4.42272727 | 0.01063796 | 0.22610483 | -0.498271012 | 12 |
| *ZBTB16* | 4.42727273 | 0.00154705 | 0.22587269 | 0.788813716 | 11 |
| *KLHL42* | 4.42727273 | 0.00154705 | 0.22587269 | -0.574373404 | 11 |
| *SIAH2* | 4.37272727 | 0.01945809 | 0.22869023 | 0.44701706 | 11 |
| *ENTPD1* | 4.02272727 | 0.11161314 | 0.24858757 | 0.789954028 | 11 |
| *OLR1* | 3.94090909 | 0.08066593 | 0.25374856 | -0.26896383 | 11 |
| *RNF41* | 4.42727273 | 0.00909091 | 0.22587269 | 0.351515646 | 11 |
| *CSF2* | 3.9 | 0.04457177 | 0.25641026 | 0.42676842 | 10 |
| *ADAM8* | 4.15454545 | 0.04963364 | 0.24070022 | 0.793910164 | 10 |
| *RNF19B* | 4.43181818 | 0 | 0.22564103 | 0.78687064 | 10 |
| *ADORA3* | 3.91818182 | 0.01742431 | 0.25522042 | 1.172369044 | 10 |
| *UBE2G2* | 4.43181818 | 0 | 0.22564103 | -0.317731004 | 10 |

FC, fold change.
